# Bioremediation of high-strength agricultural wastewater using *Ochrobactrum* sp. strain SZ1

**DOI:** 10.1007/s13205-016-0455-1

**Published:** 2016-06-23

**Authors:** Chin Hong Neoh, Chi Yong Lam, Suriati Mat Ghani, Ismail Ware, Siti Hajar Mat Sarip, Zaharah Ibrahim

**Affiliations:** 1 Centre for Environmental Sustainability and Water Security, Universiti Teknologi Malaysia, 81310 Skudai, Johor Malaysia; 2 Department of Biosciences and Health Sciences, Faculty of Biosciences and Medical Engineering, Universiti Teknologi Malaysia, 81310 Skudai, Johor Malaysia; 3 Institute of Bioproduct Development, Universiti Teknologi Malaysia, 81310 Skudai, Johor Malaysia

**Keywords:** Kraft lignin, Polyphenolic, Lignocellulolytic enzymes, Heavy metal, Palm oil mill effluent

## Abstract

The biggest agricultural sector that contributes to the Malaysian economy is the oil palm industry. The effluent generated during the production of crude palm oil known as palm oil mill effluent (POME). POME undergoes anaerobic treatment that requires long retention time and produces large amount of methane that consequently contributes to global warming. In this study, an isolated bacteria was selected based on its ability to degrade kraft lignin (KL) and identified as *Ochrobactrum* sp. The bacteria were able to treat POME (from anaerobic pond) under the aerobic condition without addition of nutrient, resulting in a significant chemical oxygen demand (COD) removal of 71 %, removal rate of 1385 mg/l/day, and 12.3 times higher than that of the ponding system. It has also resulted in 60 % removal of ammoniacal nitrogen and 55 % of total polyphenolic after 6-day treatment period with the detection of lignocellulolytic enzymes.

## Introduction

Oil palm industries contribute to the major economy of the agricultural sector in Malaysia as early as 1917. A total of 426 mills were in operation in 2011, where production of crude palm oil was 24.97 million tonnes (MPOB 2013). The earnest revenue collected from the palm oil industry has a huge contribution towards Malaysia’s development and improved standard of living. Nevertheless, the extraction of oil from the palm fruit requires large volume of water which subsequently produces wastewater high in COD values in the range of 15,000–100,000 mg/l. The palm oil mill industry has also been identified as the largest contributor to the pollution load in rivers in Malaysia, stimulating an intensive search for an alternative method which is environmentally friendly for the degradation of pollutant in POME.

With increasing government demand for environmental friendly industrial processing systems, the oil palm industry in Malaysia has been facing challenges treating the POME economically, as well as fulfilling the discharge limit stipulated by the government (Wu et al. 2010). There have been several reports on biological treatment of POME, such as on raw POME (Vijayaraghavan et al. 2007; Cheng et al. 2010) and final POME (Neoh et al. 2013b, 2015; Georgiou et al. 2016). However, to the best of our knowledge, bioremediation of POME from anaerobic pond (AnPOME) has not been previously reported. There are various methods that have been used in the treatment of POME. Liew et al. (2015) reviewed most of the polishing treatment systems for POME and suggested that membrane filtration processes, advanced oxidation processes (AOPs) with addition of hydrogen peroxide or Fenton reagent were more effective for the treatment of POME. However, the maintenance cost for MBR bioreactor was expensive due to biofouling as POME contained high concentration of suspended solids (El-Shafey and Al-Hashmi 2013; Neoh et al. 2016). On the other hand, AOPs tend to produce highly toxic by-products. Abdullah et al. (2011) developed aerobic granular sludge for treatment of diluted raw POME, and it was efficient in the removal of COD. However, the major drawback of aerobic granulation is a long start-up period of granule formation from the flocs of activated sludge (Joo-Hwa Tay et al. 2006).

Currently, the existing POME treatment mainly involves ponding systems which comprise anaerobic and facultative stages. In Malaysia, over 85 % of the mills have adopted the conventional anaerobic ponding system that was based on suspended growth of activated sludge (Zahrim et al. 2009). The anaerobic pond is one of the intermediate ponds with hydraulic retention time (HRT) of 30–80 days (Igwe and Onyegbado 2007). The anaerobic treatment is also considered as cost effective. However, anaerobic ponding system has several critical drawbacks that should be considered for further improvement, for example, extended period of HRT, solids accumulation, inconsistent nutrient removal, large land area requirement (Shilton 2006), and produced noxious odors. During the anaerobic process, a volume of 375,000,000 m^3^ methane is evolved from the open ponding systems, equivalent to 8,610,000,000 m^3^ of carbon dioxide production, which is 3.6 % of the estimated total emissions of greenhouse gases in Malaysia. POME is also one of the sources contributing to the global environmental issues specifically relating to climate change. Most of the oil palm industries in Malaysia are not equipped with biogas recovery systems due to the high capital and operating costs (Yeoh 2004). Hence, the biogas that is released into the atmosphere aggravates the effects of global warming. Since the objective of this study is to shorten the period for POME treatment, this will in turn help reduce emissions of greenhouse gases which will subsequently reduce the risks of global warming. POME contained high concentration of polyphenolic compounds which are carcinogenic, toxic, and creating unpleasant odor in water resources. Phenolic pollution has arisen as a serious problem, and there is a need for removal of phenolic in POME efficiently (Gholizadeh et al. 2013).

The main objective of this study was to treat AnPOME using single culture of bacteria. The bacteria were selected based on the ability to degrade KL. Parameters such as COD, pH, ammoniacal nitrogen, total polyphenolic compounds, lignin, color, heavy metal, and toxicity were also monitored. To the best of our knowledge, research utilizing *Ochrobactrum* sp. enzymatic systems for POME degradation has not been reported.

## Materials and methods

### Culture condition

The AnPOME obtained from the palm oil industry in Johor was stored in the refrigerator at 4 °C. For the medium preparation, AnPOME was autoclaved at 121 °C for 15 min and centrifuged at 4000 rpm, 4 °C for 15 min to remove the suspended solids to minimize the interference from measurement of optical density for bacteria growth.

### Isolation, selection, and acclimatization of bacteria

Samples from textile sludge, POME sludge (anaerobic pond and final pond before discharge), pineapple wastes, and petroleum sludge were collected and stored at 4 °C. Single colonies of bacteria from samples were obtained after incubation for 24 h at 30 °C using the streak plate method on agar medium containing anaerobic POME. KL stock solution (*M*_n_ ca. 5000, *M*_w_ ca. 28,000, Sigma-Aldrich) was prepared through dissolving it in distilled water (pH 12). Then, the KL stock solution was added in mineral salts medium that contained 0.1 % w/v KH_2_PO_4_ (QRec), 0.05 % w/v MgSO_4_.7H_2_O (Sigma-Aldrich), 0.05 % w/v NaCl (Merck), 1 % w/v glucose (Merck), and 0.5 % w/v peptone (Merck). Isolated bacteria were selected for growth in 250 ppm KL-mineral salts medium at 150 rpm (Protecth Shaker Incubator Model SI-50D), 30 °C for 4 days. Next, bacteria (10 % v/v) were selected based on the lignin reduction. To select the best bacteria for lignin reduction, bacteria were transferred into mineral salt medium supplemented with higher concentration of KL (500 ppm) in 500 ml conical flask. The bacteria that gave the highest reduction of lignin were used for the forthcoming experiments. Data from the selection of bacteria capable to reduce lignin were subject to LSD test (0.05) for the identification of significant differences between bacteria. SPSS statistics 17.0 was used to conduct LSD test and standard error for the data.

The selected bacteria were further acclimatized by, subsequently, sub-culturing the bacteria into fresh, sterilized medium supplemented with increasing concentration of AnPOME. The acclimatization process was continued until the bacteria were able to grow in 100 % AnPOME. The acclimatized bacteria were further used for treatment of AnPOME at 30 °C, 150 rpm for 6 days.

### 16SrDNA and phylogenetic analysis

Chromosomal DNA was isolated using Promega Wizard^®^ Genomic DNA Purification Kit following the standard method stated. The 16S rDNA was amplified using PCR with Taq polymerase (Qiagen) and the universal primer pair of 27F (5-AGAGTTTGATCCTGGCTCAG-3) and 1523R (5- GGTTACCTTGTTACGACTT-3) (Weisburg et al. 1991). PCR reactions were performed in 50 µl reaction volumes containing 25 µl PCR Mastermix (Qiagen), 2 µl of each of the primers (10 mM), 5 µl of the extracted DNA as the templates, and 13 µl of sterile distilled water. The PCR amplification protocol was as follows: denaturation at 92 °C for 1 min, annealing at 54 °C for 1 min, and extension at 72 °C for 1 min; all three steps were repeated for 30 cycles (Liu et al. 2005). Related sequences were obtained from the GenBank database (National Center for Biotechnology Information, NCBI) using the BLASTN search program. The 16S rRNA sequences determined and reference sequences obtained from GenBank databases were aligned using the multiple sequence alignment software CLUSTAL W ver. 1.81. Phylogenetic tree was constructed with MEGA 5.0 based on the 16S rDNA sequences of 10 strains closer to strain SZ1. The sequence was submitted to the GenBank database and assigned an accession number of KC765087.

### Analytical methods

All experiments were carried out under the aerobic condition at 30 °C with constant shaking (150 rpm). Microbial growth was monitored indirectly by measuring the absorbance at 600 nm (Buck Vis 100 Spectrophotometer). The color (ADMI unit), COD (reactor digestion method), and ammoniacal nitrogen (Nessler method) were determined by HACH DR 5000 (HACH 2005). The pH was measured using Sartorius PB-10 pH meter. Total polyphenolic compounds were quantified with Folin–Ciocalteau Reagent using gallic acid as the standard. The lignin content of the samples was estimated using KL as the standard (Neoh et al. 2013b). All experiments were conducted in triplicates.

Inductively coupled plasma-mass spectrometry (ICP-MS, Perkin Elmer Elan 6100) was applied for determination of the heavy metals in this work. The ICP-MS was operated using argon gas as carrier gas with gas flow of 0.435 l/min. AnPOME before and after treatment were filtered using 0.2 µm membrane and acidified to pH 2 with HNO_3_ for metal analysis.

### Fourier transform infrared spectroscopy (FTIR) analysis of the functional groups of the AnPOME

FTIR spectroscopy (Nicolet iS5) was used to identify the functional groups present in the samples. The POME samples before and after treatment were adjusted to pH 2 and extracted using ethyl acetate. The sample/KBr mass ratio used for the preparation of the disks was 1:200 within the IR frequency range of 100–4000 cm^−1^ at a scan speed of 16 cm/s (Lim et al. 2013).

### Enzymatic studies

The enzymatic analysis of biodegradation was performed to analyze the role of extracellular enzymes. For CMCase enzyme activities, carboxymethyl cellulose (CMC) was used as substrate, birch wood xylan for xylanase, MnSO_4_ and phenol red for manganese peroxidase (MnP), ABTS for laccase, and Azure B for lignin peroxidase (LiP) (Neoh et al. 2013a). All enzyme assays were carried out using spectrophotometer (HACH DR5000) in 50 mM sodium citrate buffer (pH 5) except for LiP which used 50 mM tartrate buffer (pH 3). CMCase and xylanase were performed at 50 °C, while LiP, MnP, and laccase were performed at 30 °C.

### Toxicity assays

The untreated and treated AnPOME were tested for their toxicity using the ToxTrak method. Toxicity assays were performed using *Bacillus licheniformis* according to the HACH instruction manual (HACH 2005).

## Results and discussion

### Isolation, selection, and identification of selected bacteria for treatment of AnPOME

A total of 23 bacteria were isolated from AnPOME agar using the streak plate method. Then, 6 bacteria that showed the ability to reduce concentration of KL (250 ppm) within 70–84 % were selected. Consequently, the selected bacteria were transferred as individual cultures into higher concentration of KL (500 ppm). However, only three out of six bacteria were tolerant to high level of KL and able to reduce concentration of KL. The strain SZ1 from textile sludge showing highest color removal (81 ± 2 %) showed significant difference (*p* < 0.05) from the rest of the bacteria, and thus were further selected for acclimatization prior to the treatment of POME. The initial color of KL was 1512 ± 45 ADMI at 500 ppm and reduced to 280 ± 20 ADMI at the end of 4 days of treatment. No change in color or lignin content was found in the control. The color reduction of KL was probably due to depolymerization of lignin polymers by bacterial ligninolytic systems (Chandra et al. 2007) or adsorption process. Even though bacteria able to adsorb colored compounds, adsorption process usually would not contribute to high degree of the decolorization process. Lim et al. (2013) investigated the desorption process from decolorization of dye by *Enterococcus faecalis*, and found out that desorption of color from bacterial pellet using 0.1 M NaOH produced approximately 11 % of color removal Strain SZ1 showed high efficiency for KL degradation as compared to previous reported studies except for co-culture of bacteria. *Bacillus* sp. Raj et al. (2007b) only showed reduction for 37 % of KL (500 ppm) and *Aneurinibacillus aneurinilyticus* (Raj et al. 2007a) showed reduction of 43 % of KL (250 ppm). Another study showed that *Bacillus subtilis* and *Klebsiella pneumonia* were able to degrade KL (800 ppm) up to 50 and 45 %, respectively (Yadav and Chandra 2015). Co-culture of the two bacteria showed a better performance in degradation of KL up to 58 % after 144 h. Shi et al. (2013a) showed that *Cupriavidus basilensis* B-8 isolated from erosive bamboo slips were able to degrade 31 % KL (2000 ppm) in 7 days. The authors also identified that MnP and laccase demonstrated the greatest enzyme activities at the third and fourth days of treatment. Shi et al. (2013b) stated that *Pandoraea* sp. B-6 were able to degrade KL (38 % removal of COD) without any co-substrate under the high alkaline conditions. Again, MnP and laccase demonstrated the greatest enzyme activities on the third and fifth days for the degradation of KL.

**Fig. 1:**
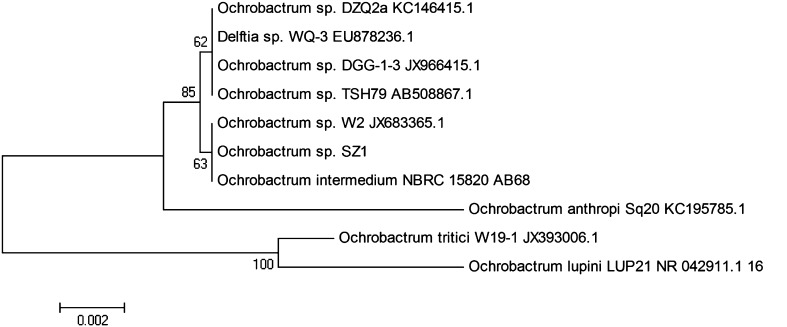
Phylogenetic tree of *Ochrobactrum* sp. SZ1

The 16S rRNA gene sequences of strain SZ1 (consisting of 1374 nucleotides) were determined, and a phylogenetic tree was constructed based on 16S rRNA sequence (Fig. 1). The 16S rRNA sequences of strain SZ1 were the most closely related to that of *Ochrobactrum* sp. and *Ochrobactrum intermedium* with a homology of 100 %. Based on the phenotypic analysis and phylogenetic characteristics, the strain could be identified as *Ochrobactrum* sp. SZ1.

To date, several studies have reported that *Ochrobactrum* species has the ability to degrade phenol (Kılıç 2009), sulphide and nitrile (Mahmood et al. 2009), tetrabromobisphenol (An et al. 2011), and N,N-dimethylformamide (Sanjeev Kumar et al. 2012). This indicated that *Ochrobactrum* species have the ability to remediate many pollutants found in the wastewater; however, there are no research and information about the potential application of this bacterial strain for the bioremediation of high-strength agricultural wastewater.

### Treatment of anaerobic POME

Analysis of autoclaved AnPOME showed color of 4747 ADMI, COD of 11707 mg/l, total polyphenolic of 916 mg/l, lignin of 721 mg/l, ammoniacal nitrogen of 256 mg/l, and BOD/COD ratio of 0.22. Samples with BOD/COD ratio less than 0.1 are considered non-biodegradable, while any value more than 0.1 is considered biodegradable (Samudro and Mangkoedihardjo 2010). In this study, BOD/COD ratio for AnPOME was 0.22 and indicated that AnPOME is biodegradable. Compared to non-autoclaved wastewater, autoclaved AnPOME showed a reduction of 10 % of COD, but no changes for the lignin and color unit.

**Fig. 2:**
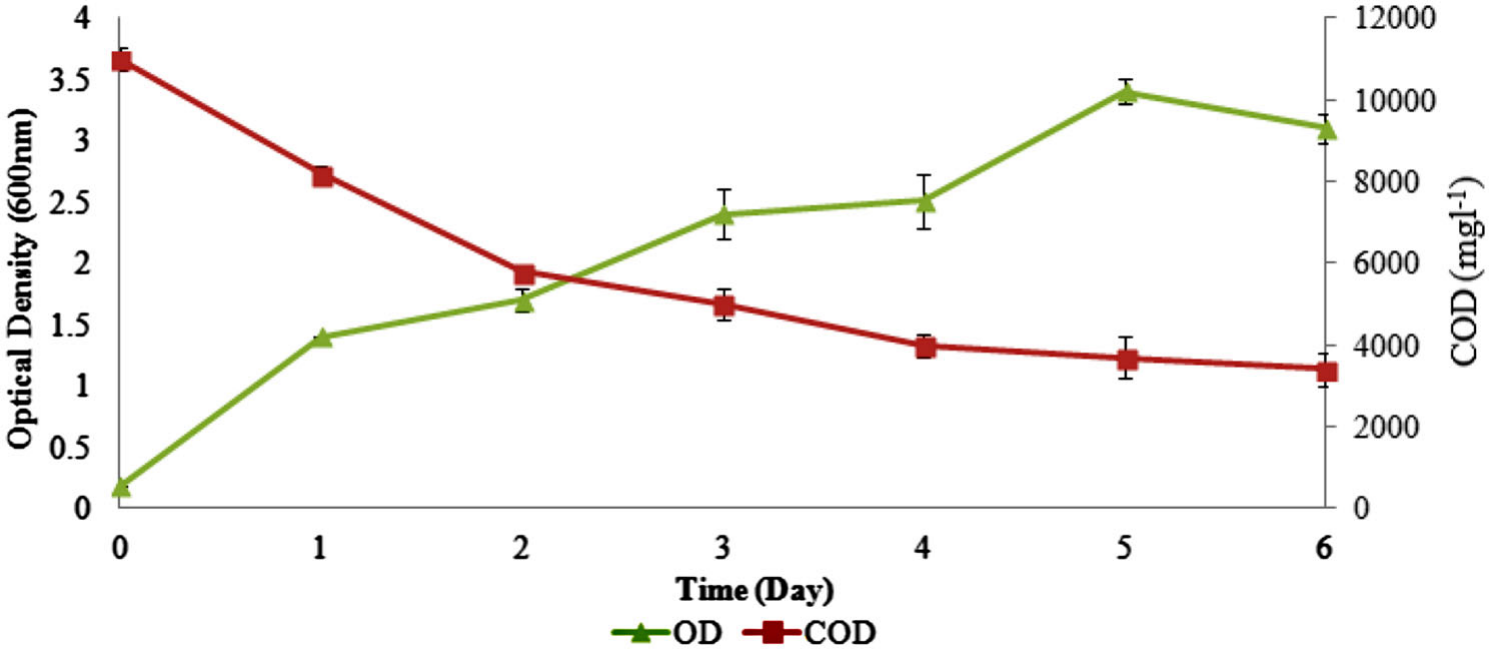
Growth profile and COD value during the treatment of anaerobic POME by *Ochrobactrum* sp.

Table 1 summarizes the reduction in effluent quality parameters when AnPOME was treated using *Ochrobactrum* sp.. The relatively high COD values of AnPOME (11,707 mg/l) was mainly due to the high organic load which may be implied to the presence of lignin, polyphenolic compounds, complex organic compounds, cellulose, or hemicelluloses found in POME. Organic compounds in AnPOME were probably used as nutrients by the bacteria, since no nutrients were added before and during the treatment process. The correlation analysis (*R* ^2^) for COD and growth of bacteria is 0.9553, and this suggested that COD removal was directly proportional to the growth of bacteria. Figure 2 shows that at the beginning, there was a significant increase in growth of the culture, reaching a maximum after 5 days of incubation, followed by a slight decline thereafter. At the same time, a significant reduction in COD occurred after 1 day of incubation. It is worth noting that the reduction of COD was maximum during the exponential phase (day 1–5) with a reduction rate of 1440 mg/l/day. The reduction rate for COD decreased to 556 mg/l/day during stationary phase suggesting the insufficient nutrient in POME. The overall treatment resulted in a significant reduction of 71 % COD removal.

**Table 1:** Reduction in effluent quality parameters using *Ochrobactrum* sp.

Parameter	Initial	Final	Percentage (%)
pH	5.3 ± 0.1	9.29 ± 0.05	−
COD	11707 ± 83	3400 ± 62	71
Ammoniacal nitrogen	256 ± 11	103 ± 5	60
Total polyphenolic compounds	916 ± 8	415 ± 10	55

All the values presented are in mg/l except pH

Values are mean of three experiments ± standard error

The AnPOME inoculated with bacteria also exhibited a decrease in ammoniacal nitrogen from 256 to 103 mg/L within 6 days of treatment. The amount of phenolic compound decreased from 916 to 415 mg/l implying that *Ochrobactrum* sp. contributed towards phenol oxidation. Another study reported that *Ochrobactrum* sp. were able to metabolize phenol through catechol by two pathways; ortho-pathway or meta-pathway (Kılıç 2009). Besides, pH of wastewater change from acidic pH to alkaline pH (9.29) is in agreement with other studies that phenol degradation was achieved more efficiently at alkaline pH levels. *Pseudomonas putida* degraded p-nitrophenol at pH 7.5 to 9.5 (Kulkarni and Chaudhari 2006), while *Orchrobactrum* sp. degraded p-nitrophenol at pH 10 (Qiu et al. 2007).

The lignin concentration in AnPOME was 721 mg/l, and there was no reduction of lignin concentration after 6 days of treatment. This may imply that lignin was not used as its carbon sources. It is important to note that bacteria that were able to degrade KL did not show the ability to degrade lignin in lignin rich wastewater, such as AnPOME. A study claimed that bacteria which are able to degrade KL could be of interest for the treatment of lignin rich wastewater, such as pulp and paper mill effluent (Raj et al. 2007b). However, results in this study were in contrast to the previous reported study, in which bacteria were able to degrade KL and they were found having no ability to degrade lignin in POME. This may be, because the chemical structure of KL which differs from natural lignin, as it undergoes various reactions, such as aryl–alkyl cleavage, strong modification of side chains, and various ill-defined condensation reactions (Chakar and Ragauskas 2004). It is well known that microorganisms exist in mixed culture due to presence of a wide range of compounds in nature. Role of co-culture, such as bacteria–bacteria (Lemaire et al. 2013) or bacteria–fungi (Liu et al. 2013) for degradation of several recalcitrant environmental pollutants have been reported. Thus, mixed culture of microorganisms may be more effective in degradation of lignin in AnPOME.

Colorless catechol is believed to be responsible for the formation of red color in POME, as the catechol will be oxidized to reddish brown melanoid pigments, derivatives of benzoquinone (Mary et al. 2012). This explains why color was found to increase by about 80 % after the treatment, which increased from 4747 ± 120 to 8507 ± 84 ADMI.

ICP-MS method is able to identify and quantify metals presence in water at trace levels. Table 2 presents the concentrations of metals in AnPOME used in this study. According to Malaysia Environmental Quality (Industrial Effluent Regulations 2009), arsenic is the only metal in POME that is slightly higher than the standard discharge limit (0.05 mg/l) for Standard A. However, it is still below Standard B (0.1 mg/l). It should be pointed out that effluent that is discharged upstream of water supply intake should meet Standard A and effluent that is discharged downstream has to meet Standard B. Addition of microorganisms to interact with dissolved metals is a way to reduce heavy metals in wastewater. Table 2 shows that the bacteria were able to reduce the concentration of selenium, aluminum, zinc, and copper from 7 to 84 %. Besides the significant removal of COD, *Ochrobactrum* sp. have the potential to resist and/or reduce the heavy metals in the wastewater. Pandey et al. (2013) stated that *Ochrobactrum* sp. were resistant to cadmium and have promised bioremediation as well as for plant growth promotion.

**Table 2:** Metal content in AnPOME stated in mg/l (Nickel and Mercury were not detected in AnPOME)

Analyte	Initial concentration	Removal percentage (%)
Aluminum	0.5552	49
Lead	0.00004	–
Cobalt	0.0033	–
Copper	0.1429	67
Zinc	1.132	84
Arsenic	0.0521	–
Selenium	0.0126	7
Cadmium	0.0001	–
Barium	0.0139	–

### Functional group identification of metabolites by FTIR spectroscopy

**Fig. 3:**
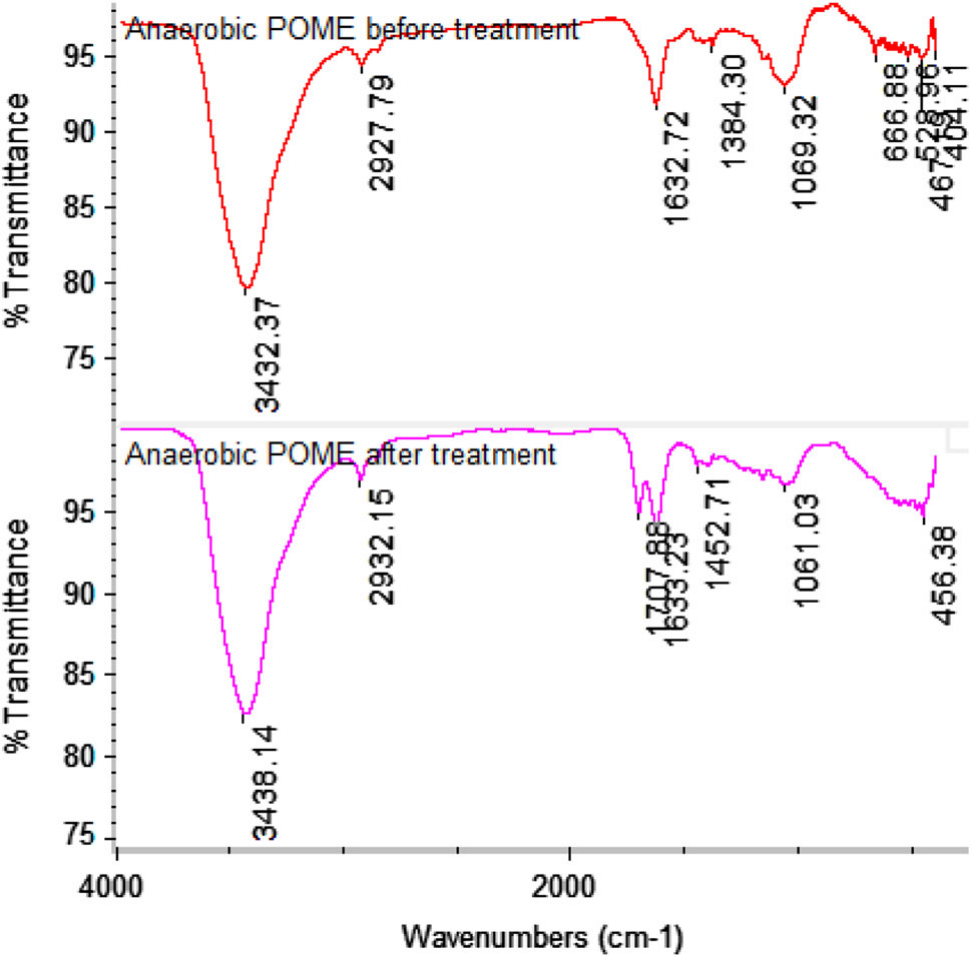
FTIR spectra of metabolites obtained from AnPOME before and after 5 days of treatment by *Orchrobactrum* sp.

The FTIR spectra obtained from AnPOME before and after 6 days of treatment revealed changes in peaks, as shown in Fig. 3. Similar and very strong peaks were observed for AnPOME before (3432.37 cm^−1^) and after treatment (3438.14 cm^−1^). These broad overlapping peaks were due to hydroxyl group or amine group presence in the AnPOME. The weak bands in the samples before (2927.79 cm^−1^) and after treatment (2932.15 cm^−1^) could be attributed to the presence of C–H sp^3^. The bands for AnPOME before treatment (1632.72 cm^−1^) and after treatment (1633.23 and 1707.88 cm^−1^) were consequences of the amide carbonyl group (C=O amide), and the shifted peak might suggest its role in degradation. The weak band for AnPOME before treatment (1384.30 cm^−1^) and after treatment (1452.71 cm^−1^) could be attributed to the presence of additional nitro groups (N=O) in AnPOME. The band for AnPOME before treatment (1069.32 cm^−1^) and after treatment (1061.03 cm^−1^) could be assigned to the –CN stretching vibration. The appearance of bands for AnPOME before treatment (666.88, 528.96, 467.19, 404.11 cm^−1^) and after treatment (456.38 cm^−1^) represents the C–N–C scissoring that is only found in protein structures (Akar et al. 2009).

### Quantitative analysis of lignocellulolytic enzymes

**Fig. 4:**
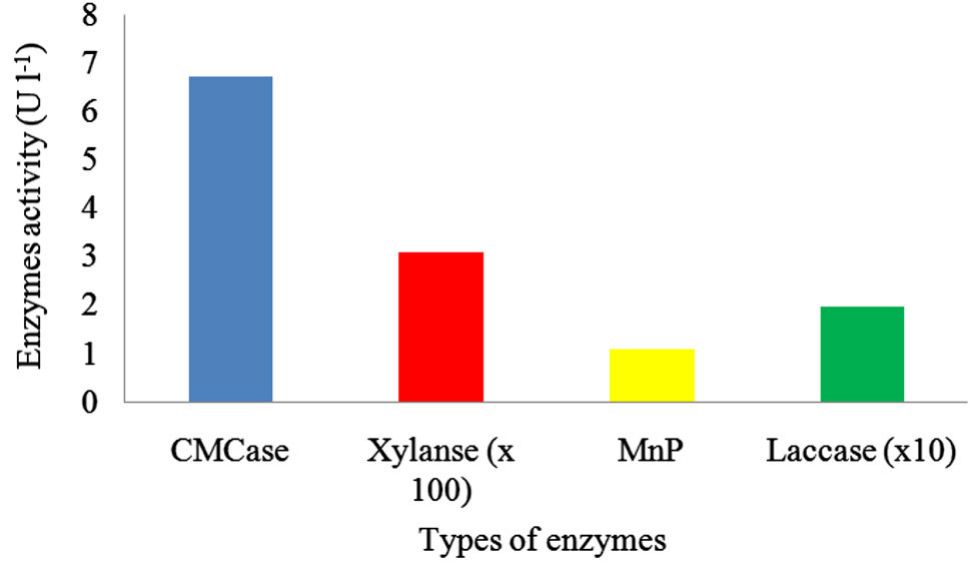
CMCase, xylanase, MnP, and laccase activities obtained in treatment of AnPOME using *Ochrobactrum* sp. (Lip was not detected in the samples)

Figure 4 shows activities of CMCase, xylanase, MnP, and laccase in biodegradation of AnPOME after 6 days of treatment. Detection of CMCase and xylanase showed the ability of *Ochrobactrum* sp. in excretion of extracellular polysaccharide hydrolyzing enzymes. LiP activity was not detected in the strain, and this might explain the failure of removal of lignin in AnPOME. The ligninolytic enzymes of *Ochrobactrum* sp. were only partial support for the higher removal of total polyphenolic compounds in AnPOME, as the enzyme activities were not detected in high level. There may be other enzymes related with the removal of total polyphenolic compounds, and the mechanisms of *Ochrobactrum* sp. in treatment of AnPOME still needs further study. The detection of lignocellulolytic enzymes indicated that biological treatment of AnPOME using *Ochrobactrum* sp. has been the greater interest in valorization of AnPOME instead of disposal of the wastewater. Besides, wastewater valorization is creating awareness among the society due to depletion of natural sources as well as increasing greenhouse gases. To the best of our knowledge, there are no reports related to the lignocellulolytic enzymes produced by *Ochrobactrum* sp. in bioremediation and biodegradation of pollutants. There was a report related using *Ochrobactrum* sp. in decolorization of malachite green through the extracellular enzymes of the strain (Vijayalakshmidevi and Muthukumar 2013).

#### Toxicity

Tox Trak method which is based on the reduction of resazurin by bacterial respiration was used in this study. Toxic substances in AnPOME can inhibit the rate of resazurin reduction. Samples that show inhibition below −10 % were considered as toxic. Table 3 shows that the percentage inhibitions for untreated and treated AnPOME are −56 and −38 %, respectively. This signifies reduced toxicity for AnPOME after treatment by *Ochrobactrum* sp. However, the treated AnPOME was still considered as toxic and a polishing stage involving physico-chemical process, such as the use of macro composite can be incorporated (Mohamad Lazim et al. 2014). It should be pointed out that the percentage inhibition (%I) results obtained are only a relative measurement and not represented as a true quantitative measurement of toxic concentration in AnPOME.

**Table 3:** Toxicity test for AnPOME and treated AnPOME using the ToxTrak method (the lower the percent inhibition, the higher the toxicity of the sample)

Sample	Percent inhibition (%)	Toxicity
Anaerobic POME	−56	Very toxic
Treated anaerobic POME	−38	Toxic

## Conclusions

The aerobic process has been demonstrated to be very suitable for the treatment of AnPOME. The identified *Ochrobactrum* sp. were able to treat AnPOME with a high efficiency to reduce COD, ammoniacal nitrogen, and polyphenolic compounds. It is important to note that the bacteria failed to degrade lignin in AnPOME, although it could degrade KL. FTIR analysis revealed the presence of hydroxyl or amine groups, C–H sp^3^, amide carbonyl, and nitro groups which could be related to consequent biodegradation of AnPOME. The high reduction of total polyphenolic compounds along with the production of lignocelluloytic enzymes indicates the suitability of *Ochrobactrum* sp. in bioremediation and valorization of various high organic strength wastewaters. Future research may include the application of *Ochrobactrum* sp. in a non-sterile condition, and co-culture with bacteria or fungus for further improvement of the degradation process. The degradation products of KL and AnPOME should also be analyzed to understand the degradation mechanisms.
